# Prevalence and Severity of Hepatic Steatosis and Fibrosis Across Glycemic Categories Among Patients Attending a Tertiary Care Hospital: A FibroScan-Based Cross-Sectional Study

**DOI:** 10.7759/cureus.116434

**Published:** 2026-09-17

**Authors:** Lisha Michel M, Kiran Kumar, Lena Sayed Taha, Amal Katalo, Koshree Nambirajan, Shreya Nair, Arwa Leelawalla, Navana Hemanth, May Thazin Win, Sinidas D S.

**Affiliations:** 1 Department of Internal Medicine, Thumbay University Hospital, Ajman, ARE; 2 Department of Internal Medicine, Gulf Medical University, Ajman, ARE; 3 Department of Obstetrics and Gynecology, Thumbay University Hospital, Ajman, ARE; 4 Department of Neurology, Thumbay Group, Ajman, ARE

**Keywords:** fibroscan, metabolic dysfunction-associated fatty liver disease (mafld), nafld and fatty liver and steatosis, prediabetes, type 2 diabetes mellitus (t2dm)

## Abstract

Introduction: Hepatic steatosis is strongly associated with metabolic dysfunction and is common among individuals with prediabetes and type 2 diabetes mellitus (T2DM). Noninvasive assessment using controlled attenuation parameter (CAP) and liver stiffness measurement (LSM) may help characterize hepatic steatosis and fibrosis risk. This study evaluated the distribution and severity of hepatic steatosis and liver fibrosis across glycemic categories in patients undergoing FibroScan assessment at a tertiary care hospital.

Methods: This retrospective cross-sectional observational study included 350 adults who underwent FibroScan assessment at a tertiary care hospital. We assessed hepatic steatosis and liver stiffness using the FibroScan Compact 530 (Echosens, Paris, France). We recorded the probe type (M or XL) where available. We performed examinations according to the manufacturer's recommendations after the required fasting period. We used LSM (kPa) to assess hepatic fibrosis and CAP (dB/m) to assess hepatic steatosis. We assigned steatosis and fibrosis categories using prespecified CAP and LSM thresholds from the clinical FibroScan protocol. We categorized participants as nondiabetic, prediabetic, or with T2DM. In addition to unadjusted chi-square analyses, multivariable ordinal logistic regression assessed associations between glycemic category and increasing steatosis and fibrosis severity, adjusting for age, sex, BMI, dyslipidemia, alcohol use, alanine aminotransferase (ALT), and relevant antidiabetic medications. We assessed the proportional-odds assumption and used multinomial logistic regression when it was not met. Adjusted odds ratios (aORs), 95% confidence intervals (CIs), and model diagnostics were reported.

Results: Among 350 participants, 82 were nondiabetic, 102 prediabetic, and 166 had T2DM. Steatosis severity differed significantly across glycemic categories (χ² = 28.96, df = 6, p < 0.001). Severe steatosis (S3) occurred in 2.4% of nondiabetic, 6.9% of prediabetic, and 18.7% of T2DM participants. After adjustment, prediabetes (aOR: 2.22, 95% CI: 1.26-3.94; p = 0.006) and T2DM (aOR: 3.58, 95% CI: 1.58-8.14; p = 0.002) were independently associated with greater steatosis severity compared with nondiabetic status. Higher BMI and ALT were also independently associated with greater steatosis severity. Fibrosis distribution did not differ significantly across glycemic groups (χ² = 3.49, df = 4, p = 0.480). In the adjusted fibrosis model, prediabetes was not significantly associated with greater fibrosis severity (aOR: 1.09, 95% CI: 0.36-3.25; p = 0.880), while T2DM showed higher but nonsignificant odds (aOR: 2.98, 95% CI: 0.81-10.96; p = 0.102). Age and ALT were independently associated with greater fibrosis severity.

Conclusion: In this tertiary care FibroScan-evaluated cohort, hepatic steatosis was increasingly severe across worsening glycemic categories, with the greatest burden observed among participants with T2DM. The association between glycemic status and steatosis remained significant after adjustment for major metabolic and clinical covariates, whereas the adjusted association between glycemic status and fibrosis was not statistically significant. These findings support using noninvasive liver assessment for metabolic risk stratification in individuals with prediabetes and T2DM, while recognizing that transient elastography does not establish a histological diagnosis of steatohepatitis.

## Introduction

Metabolic dysfunction-associated steatotic liver disease, previously known as nonalcoholic fatty liver disease (NAFLD), is a common chronic liver condition and is regarded as the hepatic manifestation of metabolic syndrome. Its progressive inflammatory phenotype, metabolic dysfunction-associated steatohepatitis, may advance to cirrhosis and hepatocellular carcinoma. Ultrasonographic studies suggest hepatic steatosis in approximately 50%-75% of individuals with type 2 diabetes mellitus (T2DM). Accordingly, the burden of MASLD appears to be especially significant among patients with insulin resistance, prediabetes, and T2DM [1].The present study aimed to evaluate the distribution and severity of hepatic steatosis and liver fibrosis across nondiabetic, prediabetic, and T2DM groups among adults undergoing FibroScan assessment at a tertiary care hospital.

## Materials and methods

Study design and population

This retrospective observational cross-sectional study included 350 adult patients who attended the outpatient department of a tertiary care hospital. The study population comprised 82 nondiabetic, 102 prediabetic, and 166 patients with T2DM. Patients aged 18-75 years with available clinical records, including alanine aminotransferase (ALT) levels, FibroScan results, body mass index (BMI), significant alcohol consumption, and medication history, were eligible for inclusion. Individuals with viral hepatitis, autoimmune hepatitis, other chronic liver diseases, a history of hepatotoxic drug use, pregnancy, or incomplete clinical data were excluded from the study. We assessed liver steatosis and fibrosis using the FibroScan Compact 530 (Echosens, Paris, France), a validated noninvasive transient elastography device widely used to evaluate hepatic steatosis and liver fibrosis. We extracted demographic, clinical, anthropometric, laboratory, and FibroScan data retrospectively from electronic medical records and analyzed them to determine the prevalence and severity of MASLD among nondiabetic, prediabetic, and diabetic patients.

Methodology

This study included adult patients undergoing FibroScan evaluation at a tertiary care hospital. Patients were categorized as nondiabetic, prediabetic, or with T2DM based on glycemic status (HbA1c <5.7% was categorized as nondiabetic, 5.7%-6.4% as prediabetic, and ≥6.5% as having diabetes mellitus). Hepatic steatosis was graded as S0-S3 (S0 < 294 controlled attenuation parameter (CAP), S1 294-310 CAP, S2 310-330 CAP, S3 331-400 CAP), and fibrosis was classified as nonfibrotic, mild, or severe based on liver stiffness measurements (kPa) using standard transient elastography criteria1 (F0 - F1 = <7 kPa, F2 = 7 - 9.5 kPa, F3 = 9.6 - 12.5 kPa, F4 > 12.6 kPa. We recorded clinical and biochemical parameters, including BMI, ALT, alcohol intake, dyslipidemia, and antidiabetic medications.

Statistical analysis

Descriptive statistics were used to summarize demographic, anthropometric, clinical, biochemical, and FibroScan variables. Categorical variables were expressed as frequencies and percentages, while continuous variables were summarized using mean ± standard deviation. Associations between glycemic category and categorical steatosis or fibrosis stages were initially assessed using Pearson's chi-square test.

For adjusted analyses, multivariable ordinal logistic regression models were constructed separately for hepatic steatosis severity and liver fibrosis severity. Steatosis stage (S0-S3) was treated as an ordinal outcome, while fibrosis stage was analyzed according to the ordered FibroScan categories. Glycemic status was the primary exposure, with nondiabetic participants serving as the reference category. Models were adjusted for age, BMI category, dyslipidemia, alcohol use, ALT, and antidiabetic medication use. Adjusted odds ratios (aORs) with corresponding 95% confidence intervals (CIs) and p values were reported.

We assessed the proportional-odds assumption before interpreting the ordinal regression models. Model convergence and overall model fit were evaluated, and multicollinearity among covariates was assessed using variance inflation factors (VIFs). Because sex was not available in the supplied dataset, it could not be included in the adjusted models. If the proportional-odds assumption was violated, multinomial logistic regression was planned as an alternative approach. All statistical tests were two-sided, and p < 0.05 was considered statistically significant.

## Results

The study included 350 adult patients who attended the outpatient department of a tertiary care hospital for final analysis. The study population comprised 82 nondiabetic, 102 prediabetic, and 166 patients with T2DM. Table 1 presents the baseline characteristics of the study population.

**Table 1:** Baseline characteristics of the study population SD: standard deviation; BMI: body mass index

Characteristic	Nondiabetic (n = 82)	Prediabetic (n = 102)	Diabetic (n = 166)	Total (N = 350)
Age, years, mean ± SD	39.7 ± 9.3	43.0 ± 8.8	47.1 ± 9.5	44.2 ± 9.7
BMI category, n (%)				
<18 kg/m²	15 (18.3)	19 (18.6)	23 (13.9)	57 (16.3)
18.5-24.9 kg/m²	37 (45.1)	32 (31.4)	77 (46.4)	146 (41.7)
25-29.9 kg/m²	28 (34.1)	43 (42.2)	60 (36.1)	131 (37.4)
≥30 kg/m²	2 (2.4)	8 (7.8)	6 (3.6)	16 (4.6)
Alcohol use, n (%)	5 (6.1)	8 (7.8)	3 (1.8)	16 (4.6)
Dyslipidemia, n (%)	53 (64.6)	83 (81.4)	153 (92.2)	289 (82.6)

The distribution of steatosis stage differed significantly among nondiabetic (n = 82), prediabetic (n = 102), and diabetic (n = 166) participants (Table 2). Pearson's chi-square test demonstrated a significant association between glycemic status and steatosis stage (χ² = 28.96, df = 6, p < 0.001). Nondiabetic participants had the highest proportion of S0 steatosis (54.8%, n = 45), whereas diabetic participants had the highest proportion of severe steatosis (S3; 18.7%, n = 31). Prediabetic participants demonstrated an intermediate distribution, including a higher proportion of S2 steatosis (23.5%, n = 24) than nondiabetic participants (7.3%, n = 6). Overall, steatosis severity differed significantly across glycemic categories, with a greater proportion of moderate-to-severe steatosis observed among participants with prediabetes and T2DM.

**Table 2:** Distribution of hepatic steatosis by glycemic status Test statistic: χ² = 28.96, df = 6, p < 0.001

Steatosis stage	Nondiabetic (N = 82)	Prediabetic (N = 102)	Diabetic (N = 166)
S0	45 (54.8%)	37 (36.3%)	59 (35.5%)
S1	29 (35.4%)	34 (33.3%)	49 (29.5%)
S2	6 (7.3%)	24 (23.5%)	27 (16.3%)
S3	2 (2.4%)	7 (6.9%)	31 (18.7%)

In contrast, the distribution of liver fibrosis assessed by FibroScan did not differ significantly across the three glycemic groups (Table 3). Pearson's chi-square test demonstrated no statistically significant association between glycemic status and fibrosis stage (χ² = 3.49, df = 4, p = 0.480). Most participants were classified as having absent or mild fibrosis (F0-F1), accounting for 91.5% (n = 75) of nondiabetic, 88.2% (n = 90) of prediabetic, and 84.3% (n = 140) of diabetic participants. F2 fibrosis was observed in 4.8% (n = 4) of nondiabetic, 8.8% (n = 9) of prediabetic, and 12.0% (n = 20) of diabetic participants. F3 fibrosis was uncommon, occurring in 3.6% (n = 3), 2.9% (n = 3), and 3.6% (n = 6) of the respective groups. No participants were classified as F4. Overall, there was no statistically significant difference in fibrosis stage across glycemic categories.

**Table 3:** Distribution of liver fibrosis by glycemic status Test statistic: χ² = 3.49, df = 4, p = 0.480

FibroScan score	Nondiabetic (N = 82)	Prediabetic (N = 102)	Diabetic (N = 166)
F0-F1	75 (91.5%)	90 (87.3%)	140 (84.3%)
F2	4 (4.8%)	9 (8.8%)	20 (12.0%)
F3	3 (3.6%)	3 (2.9%)	6 (3.6%)
F4 (cirrhosis)	0 (0.0%)	0 (0.0%)	0 (0.0%)

Multivariable analysis of hepatic steatosis

Multivariable ordinal logistic regression demonstrated that glycemic category remained independently associated with increasing hepatic steatosis severity after adjustment for age, BMI category, dyslipidemia, alcohol use, ALT, and antidiabetic medication use. Compared with nondiabetic participants, prediabetic participants had significantly higher odds of being classified into a more severe steatosis category (aOR: 2.22, 95% CI: 1.26-3.94; p = 0.006), while participants with T2DM had significantly higher odds (aOR: 3.58, 95% CI: 1.58-8.14; p = 0.002).

BMI demonstrated an independent positive association with steatosis severity. Compared with the lowest BMI category, the adjusted odds of greater steatosis severity were 1.85 (95% CI: 1.01-3.38; p = 0.046), 2.50 (95% CI: 1.36-4.57; p = 0.003), and 12.45 (95% CI: 4.44-34.94; p < 0.001) for BMI categories 2, 3, and 4, respectively. ALT was also independently associated with steatosis severity (aOR: 1.009 per unit increase, 95% CI: 1.003-1.015; p = 0.003). Alcohol use showed an inverse association in the adjusted model (aOR: 0.27, 95% CI: 0.08-0.88; p = 0.029), although the small number of participants reporting alcohol use warrants cautious interpretation.

The ordinal model converged successfully (Akaike Information Criterion (AIC) = 852.98). Assessment of the proportional-odds assumption did not identify evidence requiring replacement of the ordinal model with a multinomial model. VIFs did not demonstrate problematic multicollinearity among the explanatory variables, with the highest nonintercept VIF being 4.05.

Multivariable analysis of liver fibrosis

In the multivariable ordinal logistic regression model, glycemic category was not independently associated with increasing fibrosis severity after adjustment for age, BMI category, dyslipidemia, alcohol use, ALT, and antidiabetic medication use. Compared with nondiabetic participants, prediabetic participants had no significant increase in the odds of greater fibrosis severity (aOR: 1.02, 95% CI: 0.36-2.91; p = 0.970). Participants with T2DM had higher estimated odds of greater fibrosis severity, but this association did not reach statistical significance (aOR: 2.98, 95% CI: 0.81-10.96; p = 0.102).

Age was independently associated with increasing fibrosis severity (aOR: 1.049 per year, 95% CI: 1.013-1.086; p = 0.006), as was ALT (aOR: 1.015 per unit increase, 95% CI: 1.006-1.023; p < 0.001). Dyslipidemia, alcohol use, antidiabetic medication use, and BMI category were not independently associated with fibrosis severity after adjustment.

The ordinal fibrosis model converged successfully (AIC = 330.67). The proportional-odds assumption did not provide evidence requiring replacement of the ordinal model with a multinomial model. VIFs did not demonstrate problematic multicollinearity, with the highest nonintercept VIF being 4.05.

BMI, hepatic steatosis, and fibrosis

When hepatic steatosis was analyzed according to BMI categories within each glycemic-status group, a progressive increase in the severity of steatosis was observed with increasing BMI, particularly among prediabetic and diabetic participants. Among nondiabetic participants, the proportion with no steatosis (S0) was 73.3% in those with BMI 18.5-24.9 kg/m², compared with 48.6% among those with BMI 25-29.9 kg/m² and 53.6% among those with BMI 30-39.9 kg/m²; however, this association was not statistically significant (p = 0.500). In the prediabetic group, steatosis severity increased significantly across BMI categories (p = 0.002), with S0 decreasing from 52.6% in participants with BMI 18.5-24.9 kg/m² to 43.8% with BMI 25-29.9 kg/m² and 30.2% with BMI ≥30 kg/m². Correspondingly, moderate-to-severe steatosis (S2-S3) increased from 5.3% in the normal-BMI category to 18.8%, 41.9%, and 75.0% across the progressively higher BMI categories. A similar pattern was observed among diabetic participants, where the prevalence of S0 decreased from 47.8% in the BMI 18.5-24.9 kg/m² category to 42.9% and 25.0% in the BMI 25-29.9 and ≥30 kg/m² categories, respectively, while S2-S3 steatosis increased from 21.7% to 31.2% and 38.3%, respectively. In contrast, analysis of FibroScan fibrosis categories demonstrated no statistically significant association between BMI and fibrosis severity in the nondiabetic (p = 0.936), prediabetic (p = 0.089), or diabetic (p = 0.223) groups. Overall, these findings indicate that increasing BMI was significantly associated with greater hepatic steatosis severity in prediabetic and diabetic participants, whereas BMI was not significantly associated with FibroScan-defined fibrosis in any glycemic-status group.

## Discussion

Distribution of hepatic steatosis by glycemic status

In the present study, hepatic steatosis increased significantly with worsening glycemic status, with diabetic participants demonstrating the greatest burden of liver fat accumulation. Nondiabetic individuals were more likely to have no evidence of steatosis, whereas prediabetic and diabetic participants showed a progressive shift toward moderate and severe steatosis. These findings suggest that worsening glucose dysregulation is strongly associated with increasing hepatic fat deposition.

The close relationship between impaired glucose metabolism and hepatic steatosis is biologically plausible. Insulin resistance promotes increased lipolysis, enhanced hepatic free fatty acid uptake, increased de novo lipogenesis, and impaired β-oxidation, resulting in triglyceride accumulation within hepatocytes and progression to steatosis [2].

Notably, prediabetic individuals in our study already exhibited a substantial burden of hepatic steatosis, indicating that metabolic liver disease begins before the development of overt diabetes. Similar observations were reported by Mantovani et al., who demonstrated that hepatic steatosis is closely associated with insulin resistance and significantly increases the future risk of T2DM [3].

The diabetic group demonstrated the highest prevalence of severe steatosis (S3), further emphasizing the strong association between diabetes and hepatic fat accumulation. This finding is consistent with the systematic review and meta-analysis by Younossi et al., which reported a pooled prevalence of fatty liver disease of approximately 55% among patients with T2DM, confirming diabetes as one of the strongest risk factors for hepatic steatosis [4].

Recent international consensus statements recognize MASLD as the hepatic manifestation of systemic metabolic dysfunction and emphasize that obesity, insulin resistance, dyslipidemia, hypertension, and diabetes act synergistically to promote disease progression [5]. Our findings therefore support current recommendations advocating early liver assessment in individuals with prediabetes and diabetes to facilitate timely lifestyle intervention and optimization of metabolic risk factors.

Our findings reinforce the concept that MASLD is closely linked to metabolic dysfunction and that hepatic steatosis progresses alongside worsening glucose metabolism. The higher prevalence of moderate and severe steatosis among diabetic participants, together with the substantial burden observed in prediabetic individuals, suggests that liver fat accumulation begins early in the course of insulin resistance. This emphasizes the importance of identifying at-risk individuals before the onset of overt diabetes, when lifestyle modification and optimization of metabolic risk factors may be most effective in preventing disease progression [6].

These findings are consistent with the global epidemiological review by Younossi et al., which demonstrated that the prevalence of MASLD has risen steadily worldwide in parallel with increasing rates of obesity, metabolic syndrome, and T2DM. The review identified diabetes as one of the strongest predictors of hepatic steatosis and highlighted the growing public health burden posed by metabolic liver disease [7].

Furthermore, the 2024 European Association for the Study of the Liver-European Association for the Study of Diabetes-European Association for the Study of Obesity Clinical Practice Guidelines recognize MASLD as a multisystem disease closely associated with insulin resistance, obesity, dyslipidemia, hypertension, and cardiovascular disease. The guidelines recommend early identification of individuals with metabolic risk factors and advocate the use of noninvasive tools for liver assessment, particularly in patients with prediabetes and T2DM.

Distribution of liver fibrosis by glycemic status

Although hepatic steatosis increased significantly with worsening glycemic status, liver fibrosis did not differ significantly between the study groups. Most participants demonstrated absent or mild fibrosis (F0-F1), while advanced fibrosis (F3) was uncommon and no participant had cirrhosis.

These findings suggest that hepatic steatosis develops earlier in the disease process, whereas fibrosis reflects cumulative metabolic injury occurring over a longer duration. Progressive fibrosis is driven by chronic inflammation, oxidative stress, mitochondrial dysfunction, and activation of hepatic stellate cells secondary to persistent metabolic dysfunction.

Despite the absence of statistically significant differences, diabetic participants demonstrated a numerically higher prevalence of F2 fibrosis than nondiabetic individuals. Similar findings have been reported in previous systematic reviews, where hepatic steatosis was highly prevalent, but advanced fibrosis occurred only in a smaller proportion of patients with metabolic liver disease.

Fibrosis is now recognized as the strongest predictor of liver-related morbidity and mortality in MASLD, irrespective of steatosis severity. Consequently, identifying patients before the development of advanced fibrosis is essential to prevent progression to cirrhosis and hepatocellular carcinoma.

Current international clinical practice guidelines recommend noninvasive fibrosis assessment using transient elastography (FibroScan) in patients with T2DM because of their increased risk of advanced liver disease. The predominance of F0-F1 fibrosis in our cohort suggests that many patients were identified during the early stages of disease, providing an important opportunity for intervention through weight reduction, improved glycemic control, and management of cardiovascular risk factors.

Although fibrosis severity did not differ significantly between the study groups, the predominance of F0-F1 fibrosis suggests that most patients were evaluated during the early stages of chronic liver disease. Fibrosis is known to develop gradually following persistent hepatic inflammation and metabolic injury and therefore may not correlate directly with the degree of steatosis in cross-sectional studies. Longitudinal studies have shown that only a subset of patients with hepatic steatosis progress to advanced fibrosis, with progression influenced by factors such as duration of diabetes, obesity, genetic susceptibility, and overall metabolic control [8].

The American Association for the Study of Liver Diseases Practice Guidance emphasizes that liver fibrosis, rather than steatosis alone, is the strongest predictor of liver-related and overall mortality in MASLD. Consequently, patients with T2DM are considered a high-risk group for advanced fibrosis and should undergo noninvasive fibrosis assessment using transient elastography or validated fibrosis scoring systems, even in the presence of normal liver enzyme levels [9].

The absence of cirrhosis in our cohort represents an important clinical finding, suggesting that many patients were identified before irreversible liver damage had occurred. Early diagnosis provides an opportunity for timely intervention through sustained weight loss, improved glycemic control, management of dyslipidemia and hypertension, and the use of emerging therapies shown to reduce the risk of fibrosis progression. These findings support current recommendations advocating routine risk stratification for liver fibrosis in patients with metabolic dysfunction to prevent progression to cirrhosis and hepatocellular carcinoma [9].

Comparison with previous FibroScan-based studies

The present findings are broadly consistent with previous FibroScan-based studies demonstrating a substantial burden of hepatic steatosis among individuals with diabetes, although the absolute estimates vary according to population characteristics, referral patterns, CAP thresholds, and study methodology. In a Saudi cohort of 490 individuals with T2DM, hepatic steatosis was identified in 80.8% using a CAP threshold of ≥248 dB/m, with severe steatosis observed in 66.5%, whereas only 8.4% had liver stiffness values suggestive of fibrosis. In an Indian tertiary-care cohort of patients with T2DM and MASLD, advanced fibrosis was identified in 7.4%, while 75.8% had some degree of fibrosis by transient elastography. More recently, a large Asian-Indian cohort of 1,070 individuals with T2DM reported hepatic steatosis in 75.6% and fibrosis in 28.6%, including F3 fibrosis in 9.3% and F4 fibrosis in 9.4%. The higher proportion of advanced fibrosis in that cohort compared with the present study may reflect differences in age, duration of diabetes, metabolic burden, referral characteristics, and liver stiffness measurement (LSM) thresholds. Importantly, these studies also illustrate that CAP and LSM findings are highly dependent on the characteristics of the studied population and the thresholds applied. The present study extends this literature by comparing individuals across nondiabetic, prediabetic, and diabetic categories within the same clinical setting, demonstrating a significant gradient in steatosis severity while not demonstrating a statistically significant difference in fibrosis distribution.

Metabolic and therapeutic context

The metabolic burden underlying MASLD also has implications for therapeutic strategies. Current clinical guidance emphasizes lifestyle modification, weight reduction, physical activity, optimization of glycemic control, and management of cardiovascular risk factors in individuals with MASLD, with incretin-based therapies such as semaglutide or tirzepatide considered when clinically indicated for obesity or T2DM. Emerging evidence also suggests that glucagon-like peptide-1 (GLP-1) receptor agonists may influence alcohol-related behaviors, with a recent systematic review reporting reductions in alcohol consumption, craving, and alcohol-related outcomes in some populations, particularly among individuals with diabetes or obesity. Although alcohol use was recorded in the present study, the sample size and retrospective design do not permit assessment of the independent relationship between GLP-1 receptor agonist therapy, alcohol consumption, and liver outcomes. These observations nevertheless highlight the broader metabolic and behavioral context in which liver risk should be assessed.

Limitations

This study has several limitations. First, its retrospective cross-sectional design precludes establishing causal relationships between glycemic status and the development or progression of hepatic steatosis and fibrosis. Second, the study was conducted at a single tertiary care center, which may limit the generalizability of the findings to other populations. Third, FibroScan, although a validated noninvasive modality for assessing hepatic steatosis and fibrosis, is not the histopathological gold standard, and liver biopsy was not available for confirmation. Finally, the retrospective nature of the study limited the availability of certain clinical variables, including the duration of diabetes, lifestyle factors, and longitudinal follow-up data, which may have influenced disease severity and progression.

## Conclusions

Among adults undergoing FibroScan assessment at a tertiary care hospital, hepatic steatosis was significantly more severe across worsening glycemic categories, with the highest proportion of severe steatosis observed among individuals with T2DM. BMI was significantly associated with steatosis severity among prediabetic and diabetic participants, whereas FibroScan-defined fibrosis did not differ significantly across glycemic categories. These findings suggest that hepatic steatosis is frequently detectable in individuals with impaired glucose metabolism before or at the time of overt diabetes. However, because of the retrospective cross-sectional design and selective FibroScan-evaluated study population, the findings should be interpreted as associations within the evaluated cohort rather than evidence of disease progression. Noninvasive liver assessment may contribute to metabolic liver disease risk stratification in individuals with prediabetes and type 2 diabetes, with subsequent evaluation guided by validated fibrosis pathways and clinical risk assessment.
